# Persistent Barriers of the Gluten-Free Basic Food Basket: Availability, Cost, and Nutritional Composition Assessment

**DOI:** 10.3390/nu16060885

**Published:** 2024-03-19

**Authors:** Virginia Estévez, Juan Manuel Rodríguez, Pía Schlack, Pedro Navarrete, Karla A. Bascuñán, Victoria Núñez, Camila Oyarce, Catalina Flores, Jimena Ayala, Magdalena Araya

**Affiliations:** 1 Institute of Nutrition and Food Technology (INTA), University of Chile, Santiago 7830490, Chile; vestevez@coacel.cl (V.E.); juan.rodriguez@inta.uchile.cl (J.M.R.); pia.schlack@gmail.com (P.S.); vnunez@coacel.cl (V.N.); camoyarce10@gmail.com (C.O.); catalinafloresmora@gmail.com (C.F.); jayala@coacel.cl (J.A.); 2 Corporación de Apoyo al Celíaco (COACEL), Santiago 7830490, Chile; p.navarretemolina@gmail.com (P.N.); kbascunan@uchile.cl (K.A.B.); 3 Department of Nutrition, Faculty of Medicine, University of Chile, Santiago 8380453, Chile

**Keywords:** gluten-related disorders, celiac disease, basic food basket, gluten-free, cost, availability, nutritional quality

## Abstract

Gluten-related disorders are treated with a gluten-free diet. The “basic food basket” (BFB) consists of a list of basic foods consumed by low-income groups in society, including those lowest-cost versions within each food category. To evaluate the cost, availability, and nutritional quality of the BFB and gluten-free BFB (GF-BFB), foods were photographed, registering their cost, availability, and nutritional characteristics, in high quality and mid-range supermarkets, wholesalers, health shops, and corner shops, matching each regular BFB product with a gluten-free equivalent. Of the 1177 potential products, the selection of lowest-cost foods yielded 55 and 47 products (BFB and GF-BFB, respectively). Breads/cereals and drinks showed the highest differences (279% and 146%, respectively) while meats and sausages showed the lowest ones (18.6%). The GF-BFB cost represents 30.1% of the minimum wage, which covers the cost of 5.2 and 3.3 of the BFB and GF-BFB per month, respectively. Availability ranged between 22.7 and 42.4%. Lower availability was associated with poorer nutritional quality in the GF-BFB, which provides 5% less energy, 26% more fat, and 25% less protein than the BFB. Only 47% of gluten-free products declared their “gluten-free” condition. The results strongly suggest that the GF-BFB must be redesigned to be both gluten-free and nutritionally adequate.

## 1. Introduction

The frequency of gluten-related disorders has greatly increased in recent decades. Recent data on celiac disease estimate that the average annual increase in diagnosis is 7.5% globally [1]; to this, we currently must add non-celiac wheat sensitivity and wheat allergy, plus symptoms potentially derived from fructanes contained in wheat [1,2,3]. Although the literature has no firm prevalence figures for each of these latter conditions, our recent study reported that 8.5% of 1203 apparently healthy urban adults referred to developing symptoms after consuming gluten/wheat [4]. Because of their increasing frequency, these conditions are becoming a significant burden on healthcare systems, the affected individuals, their families, and caregivers. These illnesses are of different origin, and have diverse presentations and complications if not treated adequately, yet they share treatment, which consists of a restrictive diet that eliminates gluten [5].

*Celiac disease* originates in the small intestine when a genetically susceptible individuals consume gluten. It is characterized by autoimmune manifestations and inflammatory damage to the small intestine [6]. Until today, the mechanisms explaining their various clinical presentations are not clear. Although the disease has no cure, treatment with a gluten-free diet is effective in the vast majority of patients, provided that it is strict and permanent.

*Nonceliac wheat/gluten sensitivity.* Parallel to the increasing incidence of celiac disease, this rather new condition has become evident in the last decade [7,8]. Affected persons report intestinal and extra-intestinal symptoms after eating wheat, but they characteristically test negative both for celiac disease-specific serology and histopathology, as well as for allergy Immunoglobulin E (IgE)-mediated assays; yet their symptoms improve when gluten is eliminated from the diet. It is not certain whether gluten or some other proteins present in wheat are responsible for triggering the symptoms. Because the food market offers only gluten-free products, these patients are also treated with a gluten-free diet.

*Wheat allergy*. This is mainly described as an IgE mediated allergy, with symptoms appearing shortly after ingestion. In this condition, it is not certain which of the wheat proteins trigger the allergic reaction. Due to methodological problems, its frequency remains uncertain [4,9], and because of food market limitations, treatment of affected persons is also with a gluten-free diet.

*Fashion/trend*. This group is formed by persons following a gluten-free diet because they think it is a healthier diet or perhaps that it helps with losing weight. The most important feature in this case is that gluten-free diet represents an *option* and not *treatment*.

These conditions are different not only from the clinical point of view, but also on the consequences of eating the offending food differ. While wheat allergy is mainly described as a rapid allergy (symptoms developed within minutes to a few hours) with a risk of anaphylaxis, in CD, eating gluten tended to provoke less rapid reactions; the consequences mainly refer to symptoms that take longer to develop, and some patients may even have no clinically apparent symptoms; however, because gluten triggers autoimmune responses, each gluten ingestion potentially increases the risk of complications, including other manifestations of autoimmunity and intestinal cancer. Thus, it is agreed that maintaining gluten/wheat consumption represents a health risk for all these patients.

Thus, it is agreed that the only effective treatment for all gluten-related disorders is the gluten-free diet. This diet consists of naturally gluten-free foods (fruits, vegetables, sea foods, fish meat, poultry, legumes, nuts, and milk and dairy products) that must remain uncontaminated until consumption, and processed foods in which the production processes are controlled to prevent gluten cross contact with other ingredients, additives, colorants, etc., and subsequently avoid contamination during distribution [6]. A gluten-free diet eliminates wheat, rye, and barley grains, and their derived products, the components that trigger the clinical illness; and because the gluten-free market was historically developed for celiac disease, all persons requiring a wheat/gluten-free diet follow the restrictions given by the gluten-free products market. Gluten is a main ingredient of breads and baked goods, which are highly consumed and appreciated by the population, meaning that many patients feel that the diet is difficult and unpleasant to follow. The current fashion/trend of eating “free of”, frequently including gluten-free foods and processed products, makes the analysis more complex because, for these persons, a gluten-free diet represents a choice, and for them, eating gluten has no health consequences. It is relevant that processed foods are frequently rich in additives that may be contaminated, but they are not declared, making the selection of gluten-free products more difficult. The production of gluten-free bread is a challenge due to the important role of the gluten network in their development. Instead, cakes and cookies can easily replace wheat flour with gluten-free starches and/or flours or other gluten substitutes. Derived from these technological problems, gluten-free products often result in higher cost, and may have poorer organoleptic characteristics and nutritional quality [10].

In Chile, the state uses the concept of the family “Basic Food Basket” (BFB) to evaluate the population, identify the lower-income groups and develop specific programs aimed at supporting them. Two criteria are used for these purposes: the Social Priority Index, which uses three types of indicators: education, income, and health. With these data, the population is classified into five priority groups: high, medium high, medium low, low, and no priority. In addition, the Ministry of Social Development (MSD), which monitors family consumption habits, defines a group of 79 foodstuffs as the Basic Food Basket. They represent the foods most frequently consumed, covering a mean energy requirement of 2000 calories per person, in families formed by a mean of 4.3 persons. Cost is calculated on the basis of price rise variations applied to each product, and the MSD uses the BFB to define the “poverty line” [11]. It is well known that during the COVID-19 pandemic, everybody faced a shortage of foods, including those that have special dietary restrictions. Concurrently, the definition of BFB proper was updated and thus, today, the situation and problems posed to celiac patients and other persons suffering gluten-related disorders that depend on the GF-BFB, is uncertain. Independent of the differences incorporated to the BFB, low-income families continue depending on this set of foods. Our previous studies described that the availability of the GF-BFB was 42% that of the regular BFB, was three times more costly, and had poorer nutritional quality [12]. Data available on gluten-free foods have been conducted in different countries, and refer to characteristics of products present in the gluten-free market [13,14,15]. In this study, we focus instead on the characteristics of the list of products present in the BFB, which does not consider elements like geographical zones, brands, most popular products, nutritional characteristics, etc., with cost being the main feature determining the presence of each product in the basket. This background data led us to conduct this study, setting assessing the current status of the GF-BFB, their availability, cost, and nutritional quality as the objectives.

## 2. Materials and Methods

The study was conducted in Santiago (capital city) during October 2022. “Okto-Shop” (https://okto.shop/) is a database than maintains an updated online record of all gluten-free products available in the local market (accessed on 1 January 2020). We verified this against our own data, and obtained an initial database of products offered, identifying those present in the regular BFB and the GF-BFB; in both, the listed products represent those with the lowest cost available at the time of data recollection. The products’ availability was then confirmed by visiting the selected selling points in four districts defined by the social priority index, representing the low, medium low, high low and no priority levels. The high priority group, representing “below poverty line”, was not included due to their special characteristics and geographical features that made them unsuitable for this study. The selling points were chosen following the criteria used in previous studies [12,16], which divides them in 5 categories: high quality supermarkets, mid-range supermarkets, wholesalers, health shops, and corner shops. Table S1 shows the list of gluten-free foods that were matched with each of the foods present in the gluten-containing BFB, according to two requirements: (i) the product was labeled gluten-free (crossed wheat ear symbol or phrase), and (ii) it was the lowest-price present. Each package was photographed and characterized recording ingredients, cost, and nutritional information, as shown in the nutrition facts.

BFB and GF-BFB availability was compared by presence/absence of each listed item, gluten labeling, type of selling point, and food categories. Nutritional information appearing in the package was registered, corroborated with the Okto-Shop data, and analyzed for macro- and micronutrients as required by law. The main flours used to replace wheat and the presence/absence of micronutrient fortification labeled in gluten free “bread and cereals” (especially flours, cookies, and pasta) were also recorded. Daily calories and macronutrient intakes were contrasted against the Food and Agriculture Organization (FAO)/WHO recommendations [17]. Costs were analyzed per kilogram and per BFB’s standard monthly consumption serving, following the Ministry of Social Development criteria. Cost per person per month in the BFB and GF-BFB was calculated applying the daily standard consumption serving per person established for the BFB by the Ministry of Social Development in October 2022. For “fruits” and “vegetable” analyses, we used the cost estimated for the BFB by the Ministry of Social Development in October 2022. Descriptive statistics was used to calculate prices, intakes, and nutritional composition. Among the products included in the “(gluten-free) eating out” situation, only two restaurants were found, in one of the four districts assessed (Table S1). These places offered exclusively gluten-free menus, but none was certified as an official entity. They offered only a few of the preparations listed in the BFB, and they were located in the non-priority district, which meant a higher socioeconomic area, also known as a main gastronomic location in the city. For this reason, this section is not included in results unless specifically declared. Fruits and vegetables were not included in some analyses, because they are consumed equally by those that maintain complete or gluten-free diets. When the gluten-free equivalent of a product listed in the BFB was not found, the cost of the gluten-containing match was used for cost calculation.

## 3. Results

### 3.1. Cost (All Categories per Kilogram, BFB and GF-BFB)

A total of 18 sale points were visited in the five shop categories present in the four districts chosen. In each of them, the 55 products belonging to the BFB, and the gluten-free counterpart were searched for, resulting in 1177 potential products (726 with gluten and 451 without gluten). Selection of the lowest-cost foods yielded 55 and 47 products to be included in the BFB and GF-BFB, respectively. Table 1 shows cost per kilogram of the products in each category.

The bread and cereals cost per kilogram is 249% higher than the gluten-containing counterparts. Of the nine products included in the breads and cereals category, the highest price difference per kilogram is observed in pre/pizza (543%), spiral pasta (438%), and bread (323%). The meat and sausages category is also significantly more expensive in the gluten-free matched products (Table 1). Instead, bread pre-mix, unsweetened cookies, and instant oats are the items showing the lowest price differences, but they are still twice as expensive.

### 3.2. Cost per Person per Month (GF-BFB)

**Figure 1:**
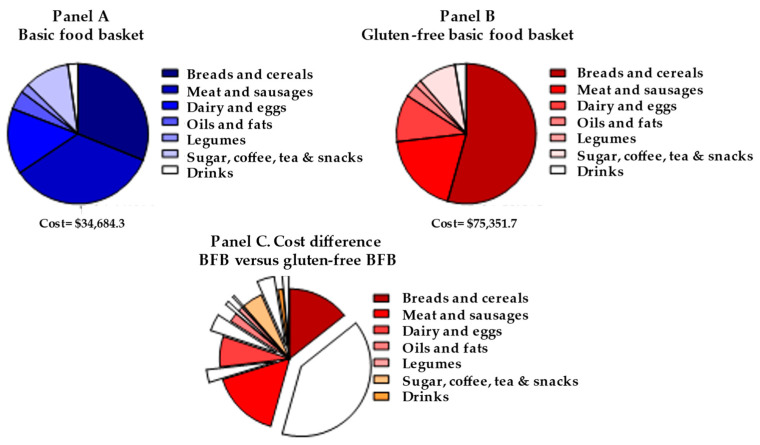
Cost of the BFB and GF-BFB (excluding fresh vegetables and meals out of home).

The total cost of the 55 products assessed, including fruits and vegetables and products belonging to “eating out” in the regular and “gluten-free baskets” was USD 85.33 and USD 132.50 per person per month, respectively, with the GF-BFB being 57% more costly. A comparison of costs excluding the fresh vegetables (no differences depending on the basket) and items belonging to “eat out” section (available only in one district assessed) are shown in Figure 1, panels A and B. All items are more expensive in the gluten-free versions, with breads/cereals and drinks showing the highest differences (279% and 146%, respectively), while meats and sausages have the lowest ones (18.6%). Breads and cereals and meat and sausages represent 31.1% and 34.4% in the BFB cost, while in the GF-BFB, these values are 48% and 21%, respectively (Figure 1, panel C).

### 3.3. BFB and GF-BFB Cost and Wages

This analysis shows that, at the time of assessment, the GF-BFB cost represents 30.1% of the minimum wage, 10.7% more than that of the gluten-containing BFB. The minimum wage covers the cost of 5.2 and 3.3 of the BFB and GF-BFB per month, respectively.

### 3.4. Availability 

Availability of the GF-BFB ranged between 22.7 and 42.4% less than that of the gluten-containing BFB. Availability per type of shop is shown in Table 2.

### 3.5. Nutritional Quality 

**Figure 2:**
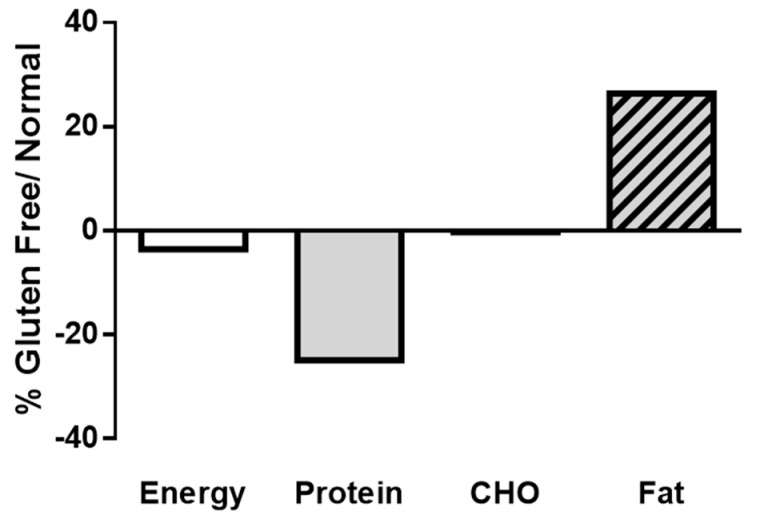
GF-BFB energy, protein, carbohydrates, and fats (in percentage) as compared to the BFB.

Considering that the Ministry of Social Development establishes the minimum daily intake required at 2000 kcal/day for an adult weighing 70 kg, the GF-BFB provides 5% less kcal per day than the gluten-containing BFB (Figure 2). The main macronutrient differences in the GF-BFB refer to the protein and fat content, which provides 25% and 26% more protein and fat, respectively, than the BFB (Figure 2). Due to the lack of data provided on the packages, it was not possible to analyze the quality of these fats (proportions of saturated, unsaturated, PUFAs, etc.). The highest differences in protein content are in the bread premix (69%) and breads (61%), despite their higher cost; the fat content is 26% greater than recommendations. The main ingredients present in breads and cereals are maize flour and starch, followed by tapioca and rice flour. Again, a lack of detail given by the nutrition facts hinders analyzing the role of the different wheat substitutes. Micronutrient content (mainly thiamine, riboflavin, niacin, and folic acid), iron and folic acid were not described in 60% and 72% of gluten-containing and gluten/free foods, respectively, also impeding further analysis. A few products declared utilizing some non-conventional flours among their ingredients (chickpea, peas, lentils, chia, and others), but the absence of details did not allow for evaluating the potential role of each of them.

### 3.6. Gluten and Allergen Declaration

Only 47% of gluten-free products declared their “gluten-free” condition; the remaining were not labeled, but were included in some private associations’ “gluten-free safe food lists”.

## 4. Discussion

This study provides relevant data about the problems faced by celiac and other persons depending on the GF-BGB after the COVID-19 pandemic. In comparison to results obtained in 2015–2016, the GF-BFB continues to be more costly and less available than the gluten-containing counterpart [12], a characteristic also reported in other countries in studies of the gluten-free products market [13,14,15,18,19,20,21,22]. Studies conducted by us during the pandemic showed a significant shortage of foods, including gluten-free foods, and that celiac persons changed their consumption habits and behaviors to maintain their safe diets [20]. However, this was not sufficient, as both the diet and treatment deteriorated, and symptoms increased during the period [23]. Availability is relevant because breads and baked goods are highly consumed, and patients often report traveling rather long distances to obtain better-quality homemade breads, consuming time and making it difficult to carry out daily life chores. An additional problem for gluten-free diet consumers is that breads and cereals are usually not purchased per portion, so these persons must be able to properly store products to keep them fresh. It is interesting that corner shops showed no differences in their availability of products with and without gluten. It can be speculated that small shops are clearly not focused on low-cost product availability; instead, they probably follow market trends focusing on the several different diets (vegan, vegetarian, others) currently followed by the population, clearly not giving priority to gluten-related health problems.

Strict comparisons of current results against the previous evaluation (2015–2016) [12,24] are difficult because some of the criteria to define the BFB were modified. This represents a limitation to this study; yet, independent of these changes, the BFB and GF-BFB remain the list of products most consumed by lower-income groups and, in this sense, some general comments are worth making. The variety of low-cost products identified in the current GF-BFB increased in comparison to the previous evaluation [12], which coincides with data, indicating that the gluten-free market has expanded [24]. However, because this study refers exclusively to the less expensive products, the analyses generally conducted in market studies should not be performed, and the role of “variety” of products available cannot be analyzed.

A gluten-free diet should not be only gluten-free, but also balanced, and sufficient in energy and nutrient requirements [25]. The nutritional value of the GF-BFB is far from satisfactory. The inadequate content of macro- and micronutrients may be associated with problems derived from the industrial production of foods, which must avoid the presence of wheat, some to the wrong choice of gluten-free products, and others to lifestyles. Several studies describe that, when following a GFD, the consumption of cereals, fruits and vegetables diminishes, while meat and its derivatives are consumed in excess [26,27,28]. On the other hand, it has been reported that the intake of packaged gluten-free products is high in children and adolescents with CD, and that the nutritional characteristics of the products they consume more frequently are poorer than their gluten-containing counterparts [29]. The evidence also shows that following GFD results in low intake of complex carbohydrates, fibers, and vitamins, mainly D, E, and the B complex (B1, B2, B6 and B9). In the case of minerals, iron, calcium, magnesium, zinc, iodine, potassium, selenium, and manganese are the main ones described as deficient in gluten-free products [30,31,32,33]. In summary, the need to evaluate the nutritional status of persons on GFD is evident. When dealing with patients with CD or other gluten-related disorders, nutritional imbalances should be characterized in detail, such that appropriate and personalized dietary guidelines can be given, promoting health and help maintaining quality of life.

Although the precise influence of the current global “eating free of” fashion/trend (including gluten-free foods) remains unclear [1,34], this trend is likely to be a relevant factor that favors the observed increase in the gluten-free foods market. The demands and expectations of groups following GFD without having a diagnosis justifying it differ from those maintaining the diet as treatment, and this could be an additional factor influencing the final characteristics of the currently available gluten-free products, and may contribute to their poorer nutritional quality and higher costs. The fact that the current study refers only to those less costly products, and that data was obtained from the packages, also limits the analyses, because we cannot assess the magnitude and potential effects that the different substitutes used may have. Finally, there is a methodological issue; there is a well-known discussion about what the safe gluten content in the gluten-free diet is (f-ESPGHAN position paper); in this and other studies of this kind, one can only refer to the products “labeled” as gluten-free, but gluten is not quantified, and the uncertainty that this implies represent an additional limitation to these studies.

**Figure 3:**
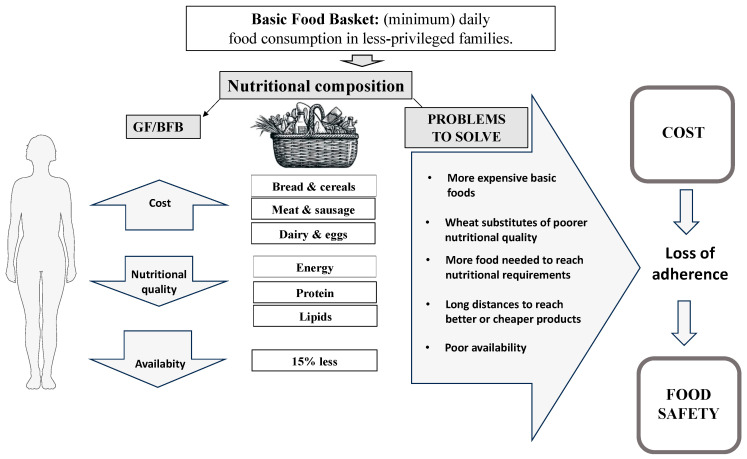
Challenges to be solved to maintain a good quality GF-BFB.

It was interesting to find that a few products have incorporated unconventional flours, like some made of legumes or nuts, a fact that suggests an emerging awareness regarding to the need to improve the nutritional quality of gluten-free foods; this should be encouraged, as shown in Figure 3.

The relation between results presented here and the information available in the literature deserves a special comment. It is relevant that this study is based on a governmental list built on the basis of large population surveys, on which subsequently different types of criteria are applied to calculate poverty indicators. The interest is focused on the global characteristics of the GF-BFB and the consequences that consuming this group of foods may have on the nutrition of low-income persons and families. This contrasts with the vast majority of published studies on gluten-free foods (market studies), which are based on ad hoc consumption preference surveys, geographical availability, global market data, etc., all of which do not consider what should be available in the market to maintain good nourishment in the lower-income groups. The fact that components of these baskets include only the cheapest products makes it advisable to not compare results to data obtained in the gluten-free products to the general market. Additionally, our results agree with market studies describing that the gluten-free products offered are, in general, more costly, less available, and with poorer nutritional characteristics [1]. A relevant result presented here is that choosing wrongly among the components of GF-BFB may lead to a significant increase in both the nutritional and social risk of these persons. From another point of view, since persons following GFD without a diagnosis justifying it seem to continuously increase [35,36], they should be advised of the risks that the diet’s nutritional characteristics imply and, consequently, GFD should be advised only to persons that need it as treatment. It is not known what potential long-term effects the nutritional deficiencies described may have on health. Additionally, if the diet is followed as an option, it should be supervised by a professional trained in restrictive diets.

Targeted interventions aimed at persons suffering gluten-related disorders and the gluten-free diet would help with promoting healthy eating behaviors, impede quality of life deterioration, and potentially minimize the anxiety described by persons that must maintain restrictive diets in modern societies [37].

## 5. Conclusions

It is well known that to keep a strict GFD is hard for everybody. Results of this study show that it is considerably more difficult for lower income families because the GF-BFB is less available and more costly. The poorer nutritional quality of the GF-BFB found is especially relevant because it adds nutritional risk to a group of patients that is already at higher nutritional risk due to the illness they suffer.

## Figures and Tables

**Table 1:** Cost per kilogram of products in BFB and GF-BFB in each food category.

Product	BFB * Price/Kilogram	GF-BFB ** Price/Kilogram	GF-BFB BFB	%∆	Chi 2
Bread and cereals	20,113	58,992	2.93	193.30	<0.05
Meat and sausages	83,706	97,404	1.16	16.36	<0.0001
Dairy and eggs	27,421	37,495	1.37	36.74	<0.05
Oils and fats	12,480	14,563	1.17	16.69	NS
Legumes	3220	5122	1.59	59.07	NS
Sugar, coffee, tea, snacks	45,863	95,508	2.08	108.25	NS
Drinks	12,686	28,995	2.29	128.56	NS
Total	205,489	338,079	1.65	64.52	

BFB *: Basic Family Basket. GF-BFB **: Gluten-free Family Basket.

**Table 2:** Availability of products contained in the BFB and GF-BFB in the five types of shops assessed.

Store/Supermarket	Availability (%)		
	BFB *	GF-BFB **	∆ BFB/GF-BFB
**Quality supermarket**	99.1	76.4	22.7
**Mid-range supermarket**	94.1	60.9	33.2
**Wholesale**	83.6	41.2	42.4
**Health shops**	59.1	21.8	37.3
**Corner shop**	20	20	0

BFB * = Basic Food Basket. GF-BFB ** = Gluten-free Basic Food Basket.

## Data Availability

The raw data supporting the conclusions of this article will be made available by the authors on request.

## Supplementary Materials

The following supporting information can be downloaded at: https://www.mdpi.com/article/10.3390/nu16060885/s1, Table S1. List of categories and foods included in the GF-BFB, per portion and calories per day.
