# Supplementary material for: Persistent Barriers of the Gluten-Free Basic Food Basket: Availability, Cost, and Nutritional Composition Assessment
Source: Nutrients. 2024 Mar 19;16(6):885. doi: 10.3390/nu16060885 (PMC10974532; doi:10.3390/nu16060885)
Supplement: Supplementary file 1 [file nutrients-16-00885-s001.zip › nutrients-2850513-supplementary.pdf]

Table S1. List of categories and foods included in the GF-BFB, per portion and calories per day.

	Food	Portion*	Portion per day	Calories per day
Bread and Cereals	Rice	g	22.2	81.1
	Oatmeal	g	5.1	0.1
	Pasta	g	20.2	26.2
	Sweet biscuits	g	1.1	5.4
	Salted biscuits	g	0.5	1.9
	Wheat flour (plain)	g	10.2	37
	Bulk bread	g	151.2	436.9
	Prebaked pizza dough (family size)	g	0.8	0.1
	Store-bought ready to eat cake (15-20 servings)	g	7.2	27.2
Meat and Sausages	Sirloin butts	g	0.3	0.4
	Minced turkey meat	g	1.2	1.4
	Minced beef meat (10% fat)	g	4.9	8.5
	Fresh mussels	g	2.7	2
	Pork chop	g	3.9	11.2
	Pork ribs	g	1.8	5.2
	Ham	g	9.4	25.6
	Canned Jack mackerel	g	18.8	52.6
	Sausages (*longaniza*)	g	4.2	13.5
	Fresh or frozen sea bass	g	8.1	6.2
	Veal spread	g	2.9	12.4
	Chicken breast	g	4.7	9.2
	Whole chicken	g	20.4	36
	Pork meat	g	4	5.2
	Fresh or frozen lamb meat	g	0.2	0.2
	Turkey sausage	g	2.3	5
	Traditional pork sausage	g	0.2	1.1
	Canned sea food	g	0.3	0.3
	Chicken drum	g	15.3	37.9
Dairy and eggs	Hen egg	g	13.1	21
	Instant powdered whole milk	g	3.2	15.8
	Whole cow's milk	ml	73.7	44.2
	Fresh cheese or soft cheese	g	0.9	1
	Cream cheese	g	0.3	1.1
	Gouda cheese	g	4.3	15.1
	Bulk yogurt (1L)	mL	20.8	17.8
Oils and Fats	Vegetable oil	mL	15.7	140.7
	Salted butter	g	1.2	8.8
	Margarine	g	3.6	25.5

Legumes	Beans	g	13.1	42.8
	Lentils	g	1.7	5.7
Sugar, Cofee, Tea & Snacks	Peanuts (salted)	g	0.4	2.4
	Sugar	g	28.8	114.6
	Chocolate	g	0.9	5
	Candies	g	1.9	7.5
	Ice-cream (family size, one flavor)	mL	9.3	13.1
	Tomato sauce	g	20.1	16.9
	Coffee substitute	g	1	0
	Tea leaves	g	1.8	0
Drinks	Mineral water	mL	9.4	0
	Soft drinks	mL	46.9	17.7
	Energy drink	mL	0.1	0
	Isotonic drink	mL	0.3	0
	Fresh fruit juice	mL	4.5	2.5
	Fruit flavored drink	mL	0.1	0.1
	Powdered juice mix	g	1.3	4.9
Eating Out Products	Hot dog	g	6.4	18
	Potato chips	g	0.8	1.3
	Regular black tea (for breakfast)	g	1.3	0.3
	Breakfast breads and pastries	g	0.1	0.5
	Starter (salad o soup for lunch)	g	0	0
	Dessert (for lunch)	g	0	0
	Combo fast food	g	0.7	1.5
	Toasts (avocado or butter or jam for breakfast)	g	0	0.1
	Ham and cheese or steak and cheese sandwich (teatime)	g	0	0.1
	Whole roasted chicken	g	2	3.7
	Empanada (meat pie)	g	1.5	2.9
	Daily special (for lunch)	g	4.5	5.1
	Main course (for lunch)	g	0.8	1.5
Fruits and Vegetables	Banana	g	110.2	100.5
	Apple	g	51.1	30.2
	Lettuce	g	25.2	3.3
	Pumpkin	g	14.2	4.5
	Lemon	g	15.7	4.6
	Avocado	g	12.5	20.1
	Tomato	g	44.6	9.4
	Carrots	g	6.3	2.7
	Onions	g	17.5	6.6
	Corn (frozen)	g	5.7	4.6
	Potato	g	121.5	218.5
